# Vectors of arboviruses in the state of São Paulo: 30 years of *Aedes aegypti* and *Aedes albopictus*

**DOI:** 10.11606/s1518-8787.2019053001264

**Published:** 2019-09-18

**Authors:** Dalton Pereira da Fonseca, Lígia Leandro Nunes Serpa, Gerson Laurindo Barbosa, Mariza Pereira, Marcia Moreira Holcmam, Júlio Cesar Voltolini, Gisela Rita Alvarenga Monteiro Marques

**Affiliations:** I Superintendência de Controle de Endemias. Departamento de Controle de Vetores. São Paulo, SP, Brasil; II Universidade de Taubaté. Faculdade de Ciências Biológicas. Taubaté, SP, Brasil

**Keywords:** Aedes, Mosquitos Vetores, Vetores de Doenças, Análise Espacial, Controle de Vetores

## Abstract

**OBJECTIVE:**

To describe the infestation of the municipalities of São Paulo by the vectors *Aedes aegypti* and *Aedes albopictus,* characterize seasonality and analyze average temperatures and larval densities.

**METHODS:**

We used maps with information on the infestation of municipalities between 1986 and 2015. The analysis of larval density of the species by the Wilcoxon test used the Breteau index values for *Ae. aegypti* and *Ae. albopictus* obtained from the Superintendency for Endemic Diseases Control database. In the seasonal description, arithmetic means of each vector were calculated by month and year. Mean temperature analyses were presented on maps with color gradients.

**RESULTS:**

The state of São Paulo is currently almost totally infested, with co-occurrence of species in 93.64% of the municipalities. The seasonality analysis showed the first quarter as the most favorable period for larval abundance. The increase of mean temperatures in geographical areas coincided with the temporal trajectory of *Ae. aegypti* territorial expansion. The mean larval density found was higher for *Ae. aegypti* than for *Ae. albopictus* (p = 0.00).

**CONCLUSIONS:**

Initially, these Culicidae occupied distinct and opposing areas. Over time, however, co-occurrence showed how great their capacity for adaptation is, even in the face of different social and urban conjunctures. The increase of the mean temperature contributed to *Ae. Aegypti* ’s geographic expansion, as well as to the clearly seasonal profile of both species. In general, larval infestation by *Ae. aegypti* prevailed, which evidenced its competitive superiority. These data provide a better understanding of the dynamics of arboviral transmission in the state of São Paulo and can be used in vector surveillance and control.

## INTRODUCTION

*Aedes* ( *Stegomyia* ) *aegypti* and *Aedes* ( *Stegomyia* ) *albopictus* are major vectors of viruses that cause emerging and reemerging diseases, such as dengue, Zika, chikungunya and yellow fever, and have a wide geographical distribution. The geographic spread of these mosquitoes in the world is influenced by different factors and has been accompanied, in some places, by decrease in their abundance, elimination of the other vector, or coexistence in extensive regions of America^1^ . In Brazil, these species were introduced at different times: *Ae. aegypti* in the colonization period and *Ae. albopictus* at the end of the 20th century^2 , 3^ .

In the state of São Paulo (SP) in the early 1980s, the presence of Ae. Aegypti was detected in the port region of Santos. Five years later, an extensive entomological survey identified household infestation in nine municipalities, all located in the northwest region of the state^3 , 4^ . In 1987, outbreaks of *Ae. albopictus* were recorded in the eastern region of SP^5^ . The detection of these species triggered specific control actions; however, efforts remain focused in an attempt to keep the infestation rates of these vectors low^6^ .

The spread of these mosquitoes was probably favored by the increased circulation of people and the intensification of commercial activities, factors that aggravate the risk of arboviruses, even in hitherto unaffected regions^7^ . This picture may have been further intensified by the effect of climate change, which in turn impacts on the distribution of these transmitters^8^ .

The presence of these mosquitoes in São Paulo municipalities has been identified in recent years in a growing and accelerated way, a profile that poses difficulties for the programs and actions to control these vectors. This reinforces the importance of entomological research, which can help define the spatial boundaries of the transmission of various arboviruses and better understand their ecological and occupation processes in the territorial space over time. Thus, knowledge of the spatio-temporal distribution and abundance of these vectors is an important objective of entomological surveillance, since it allows monitoring their population behavior, essential for planning public health actions. Accordingly, we here intended to describe *Ae. aegypti and Ae. Albopictus* in São Paulo municipalities, assuming that their population densities are different.

## METHODS

This is a descriptive and retrospective study of the infestation of the state of São Paulo by *Ae. aegypti* and *Ae. albopictus* from 1986 to 2015 *.* The study area is the most populous state of the federation, with approximately 44.85 million residents and a population density of 168 inhabitants per square km. Located in the Southeastern region of the country, it borders the states of Paraná, Mato Grosso do Sul, Minas Gerais and Rio de Janeiro. It is one of the most important development poles of the Southern Hemisphere, representing 3% of the Brazilian surface, although its economic influence far exceeds its territorial limits^9^ . The climate classification covers seven types, with humid climate prevailing. The dominant type is characterized by the tropical climate of altitude, with rains in the summer, drought in the winter, average annual temperature of 20°C to 22°C, and average temperature of the hottest month above 22°C^10^ .

The geographic space of São Paulo has undergone, over the last years, socioeconomic transformations accompanied by an intense process of redistribution of the human population, which resulted in a regionally differentiated concentration^11^ . The entomological indicator analyzed was the Breteau index (BI), adopted to follow the surveillance and control activities, which allows to obtain levels of larval infestation by *Ae. Aegypti* and *Ae. albopictus* for each municipality^6 - 12^ .

$$\mathrm{BI}\;=\;\frac{\mathrm{number}\;\mathrm{of}\;\mathrm{positive}\;\mathrm{containers}\;\mathrm{for}\;\mathrm{the}\;\mathrm{presence}\;\mathrm{of}\;\mathrm{species}}{\mathrm{No}.\;\mathrm{of}\;\mathrm{properties}\;\mathrm{searched}}\;x\;100$$

The data were obtained from the online information system of the Superintendency for Endemic Diseases Control (SUCEN), shared by the state and its municipalities. BI values measure the breeding sites by counting the containers with larvae of each species in groups of 100 surveyed properties. The value indicates the intensity of household infestation, which allows to estimate the density.

The description of the infested municipalities for each species, in time and space, was presented in annual maps. Infested municipality is one in which the species is established, that is, present even after controlling actions, with records of its development and reproduction, harboring in its extension immature forms of these vectors^3^ .

To characterize seasonality and analyze population densities, we calculated the arithmetic means of BI values of each species by month and year of the study period. The data were analyzed using the Bioestat 5.3 software, and compared by the Wilcoxon matched pairs test or Wilcoxon signed-ranks test. Values of p < 0.05 were considered significant.

The mean temperatures in the state of São Paulo were analyzed from data from the meteorological stations of the Meteorological Database for Teaching and Research of the *Instituto Nacional de Meteorologia* (National Meteorological Institute^13^ ). The data are presented on maps for the years 1986, 1990, 1995, 2000, 2005, 2010 and 2015. The colors represent the different temperature gradients, with the highest indicated by the dark red tone and the lowest by the lighter tone.

## RESULTS

The historical series of 30 years of infestation of the municipalities of São Paulo by *Ae. aegypti* or *Ae. albopictus* is shown in Table 1 . Between 1986 and 1996, the number of municipalities increased from 572 to 645, a figure that remains to the present day.

**Table 1:** Distribution of the number of municipalities infested by *Ae. aegypti* or *Ae. Albopictus* . State of São Paulo, Brazil, 1986–2015

Número de municípios						

Year	Existing	Infested by *Ae. aegypti*	Infested by *Ae. albopictus*	Mixed infestation*	Total infested	% with infestation
1986	572	65	0	0	65	11.36
1987	572	133	6	0	139	24.30
1988	572	186	19	0	205	35.84
1989	572	227	23	6	256	44.76
1990	572	257	38	35	330	57.69
1991	582	282	48	48	378	64.95
1992	625	262	91	88	441	70.56
1993	625	227	147	159	533	85.28
1994	637	170	174	236	580	91.05
1995	637	162	178	255	595	93.41
1996	645	105	179	321	605	93.80
1997	645	77	182	368	627	97.21
1998	645	74	165	389	628	97.36
1999	645	73	154	401	628	97.36
2000	645	72	150	406	628	97.36
2001	645	72	142	414	628	97.36
2002	645	48	132	450	630	97.67
2003	645	42	126	462	630	97.67
2004	645	40	124	466	630	97.67
2005	645	40	117	473	630	97.67
2006	645	40	107	483	630	97.67
2007	645	39	96	495	630	97.67
2008	645	39	87	504	630	97.67
2009	645	37	74	519	630	97.67
2010	645	37	59	535	631	97.83
2011	645	39	36	566	641	99.38
2012	645	38	34	569	641	99.38
2013	645	38	22	581	641	99.38
2014	645	36	14	591	641	99.38
2015	645	35	4	604	643	99.69

*Infested by both species.

Infestation by *Ae. aegypti* started in 1986, while by *Ae. Albopictus* in 1987, in distinct and opposing geographic areas. In the first year, the dispersion of these *Aedes* reached, respectively, 133 (23.25%) and 6 (1.05%) of the municipalities existing at the time. In the following years, the number of municipalities infested by one or the other species increased, with a higher spread and growth of *Ae. aegypti* than of *Ae. albopictus* . The number of municipalities infested in the first half of the study (1986 to 2000) was greater than in the second half. In 1994, the infestation reached the level of 91.05%. In the last year of the study, 2015, almost the entire territory of São Paulo (99.69%) was infested.

The co-occurrence of these *Stegomyia* , that is, mixed infestation, was found in six municipalities in 1989, rising to 37.05% in 1994, at which time the number of municipalities infested exclusively by *Ae. albopictus* also increased, but in a geographical area other than that occupied by *Ae. aegypti* . Although mixed infestation has changed over time and from region to region, it began to dominate the state’s geographic space and was reported in 604 (93.64%) of the existing municipalities. Of the 41 remaining municipalities, 35 are still exclusively infested by *Ae. aegypti* and six by *Ae. albopictus.* Located in different geographic areas, the municipalities infested by *Ae. aegypti* were in the midwest of São Paulo, while those infested by *Ae. Albopictus* were in the Greater São Paulo and Paraíba Valley region. Only the municipalities of Campos do Jordão, in the Paraíba Valley region, and Ribeirão Grande, in the Sorocaba region, did not register infestation by these species.

**Figure 1:**
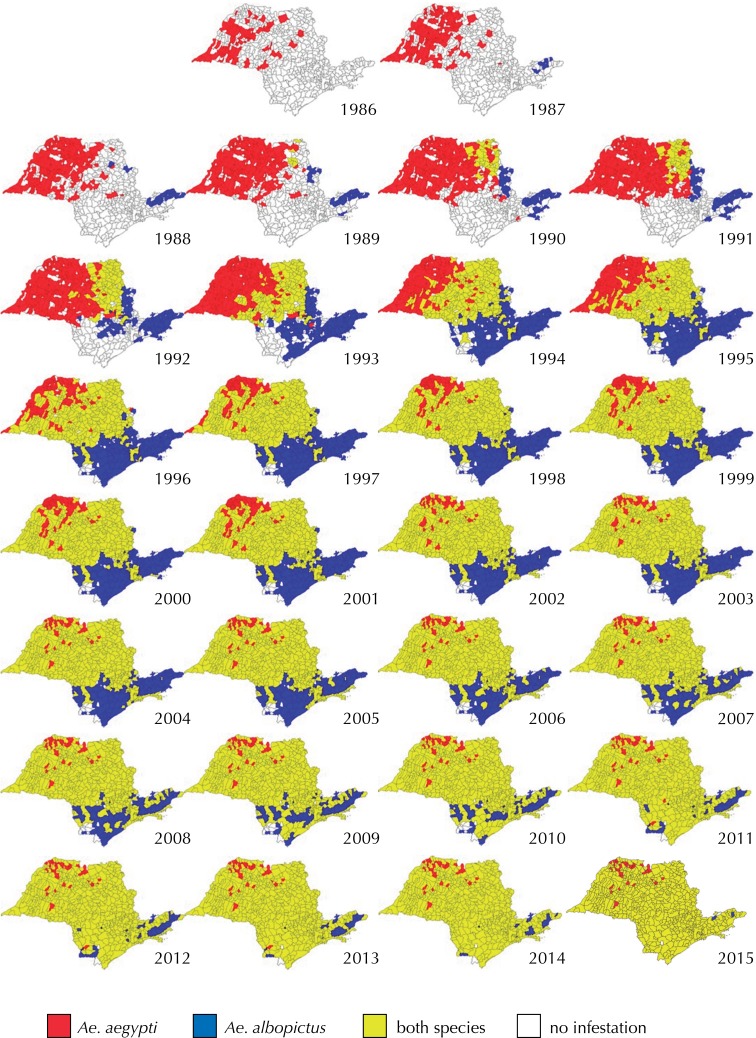
Municipalities infested by *Ae. aegypti,* by *Ae* . *albopictus* and by both species. State of São Paulo, 1986–2015.

Figure 1 shows the geographical trajectory of this infestation. It is seen that the occupation did not take place homogeneously in time and space, but there was a rapid geographic expansion. In the first year, 1986, *Ae. aegypti* is established in the northwest region, upper half of the state. In the following year, 1987, *Ae. Albopictus* was first recorded in municipalities located on the banks of the Presidente Dutra highway (BR-116), in the RJ-SP direction. This area is called *Vale do Paraíba* (Paraíba Valley), in the eastern region of SP, opposite to that occupied by *Ae. aegypti* . In the sequence, in 1988, that species was established in a few municipalities in the northeastern part of the state. The co-occurrence of these species was recorded in the north-northeast region, until then occupied only by *Ae.albopictus* .

In the years that followed, there was a great expansion of the infested area, so that in the first half of the 1990s, three patterns in the geographical expansion of these species could be differentiated. Further to the northwest of the state, the infestation occurred by *Ae. Aegypti* ; to the south-southeast, by *Ae. albopictus* ; and in the northeast to southwest direction, a central band, with mixed infestation.

Chronologically, co-occurrence advanced faster in the northwest-west direction than in the south-southeast region, hitherto occupied only by *Ae. albopictus* . This picture did not last long, and from 2001 on *Ae. aegypti* has spread through most of the Paraíba Valley region, the last of the state to be infested by this species. As can be seen, in 2015, few municipalities remained free from the co-occurrence of the two species.

**Figure 2:**
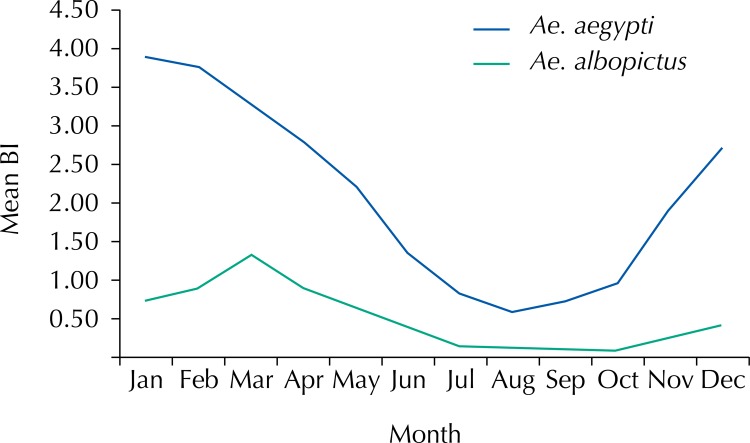
Monthly distribution of the Breteau index (BI) mean values for *Ae. Aegypti* and *Ae. Albopictus.* State of São Paulo, 1986–2015.

Figure 2 shows the mean monthly BI values for *Ae. aegypti* and *Ae. albopictus* over 30 years of survey. A similar seasonal behavior is observed, but with different larval density values. Although with different frequencies, there are records of both species in every month of the year. The results showed that vectors most abounded in the first trimester, summer months (January to March). In the second quarter (April to June), the density indexes of both species fell, and from July to October *Ae. albopictus* shows quite reduced values. On the other hand, *Ae. aegypti* reduced its density until August (BI values close to 1.0), but resumed its population growth in the other months of the year (October to December), with average rates close to 3.0.

**Figure 3:**
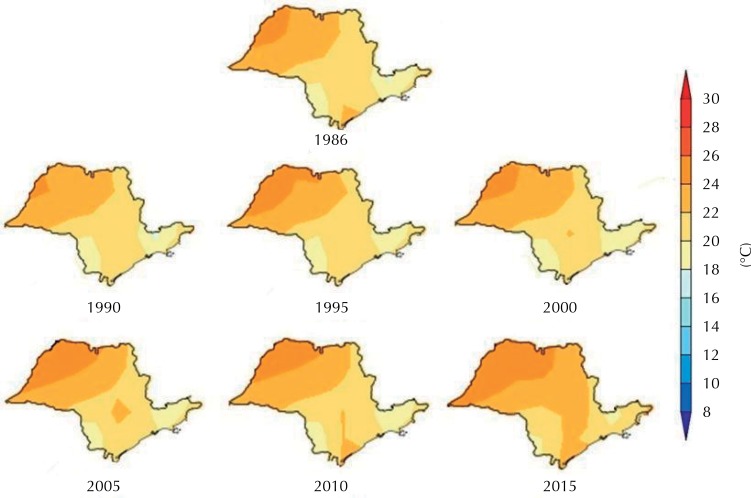
Average temperature profile by year. State of São Paulo, Brazil, 1986, 1990, 1995, 2000, 2005, 2010 and 2015.

Figure 3 shows the profile of the mean temperature in degree Celsius (°C) for the state of São Paulo. The seven maps show the chronological sequence of expansion of the geographic range with higher mean temperatures. Note that this elevation is growing over the years, moving to the interior of the state, from the plateau alignment, and to the coast, near the sierras of Paranapiacaba, do Mar and da Mantiqueira.

Table 2 shows the mean BI values. Notice that they varied according to year and species; however, *Ae. aegypti* indicators were always higher than those for *Ae. albopictus* . During the study period, 416 larval density evaluations were performed per year in average, as a result of 26,910,205 households surveyed.

**Table 2:** Annual distribution of the Breteau index (BI) mean values and percentage of containers with each species. State of São Paulo, Brazil, 1986–2015

Year	BI *Ae. aegypti*	BI *Ae. albopictus*	BI total	% of containers with *Ae. aegypti*	% of containers with *Ae. albopictus*
1986	2.28	0.01	2.29	99.56	0.44
1987	2.18	0.01	2.19	99.54	0.46
1988	2.73	0.19	2.92	93.52	6.48
1989	2.96	0.10	3.06	96.68	3.32
1990	3.60	0.19	3.79	94.98	5.02
1991	1.84	0.21	2.05	89.59	10.41
1992	2.35	0.44	2.79	84.20	15.80
1993	2.76	0.02	2.79	99.18	0.82
1994	2.40	1.61	4.02	59.87	40.13
1995	2.30	1.55	3.85	59.77	40.23
1996	3.68	1.38	5.07	72.68	27.32
1997	3.17	1.03	4.20	75.48	24.52
1998	3.42	1.42	4.83	70.72	29.28
1999	2.39	0.91	3.30	72.52	27.48
2000	3.43	1.53	4.96	69.25	30.75
2001	2.34	0.97	3.31	70.71	29.29
2002	1.15	0.45	1.60	72.14	27.86
2003	1.23	0.39	1.62	75.96	24.04
2004	1.54	0.40	1.95	79.28	20.72
2005	1.98	0.33	2.30	85.81	14.19
2006	1.59	0.22	1.80	87.96	12.04
2007	1.38	0.20	1.58	87.31	12.69
2008	1.02	0.17	1.19	85.75	14.25
2009	1.41	0.14	1.54	91.09	8.91
2010	0.98	0.21	1.19	82.50	17.50
2011	0.97	0.15	1.11	86.82	13.18
2012	1.08	0.11	1.19	90.65	9.35
2013	1.26	0.14	1.40	90.30	9.70
2014	1.05	0.09	1.13	92.38	7.62
2015	1.11	0.12	1.23	89.87	10.13
Mean	2.05	0.48	2.54	83.54	16.46
Standard error	0.16	0.11	0.23	2.06	2.06

The mean BI of the 30 years of study was 2.05, with standard error (SE) = 0.16, and 0.48 (SD = 0.11) for *Ae. aegypti* and *Ae. albopictus* , respectively. Of the total number of containers surveyed, the presence of the first and second species was recorded in 83.54% and 16.46%, respectively. The comparison of *Ae. Aegypti* and *Ae. Albopictus* mean BI values showed a significant statistical difference (n = 30, T = 0.00, Z = 4.78, p < 0.001), indicating that the mean larval density was significantly higher for the former than for the latter, 4.27 times as high, translating into the greater percentage of positive containers for the presence of immature forms of *Ae. aegypti* .

## DISCUSSION

The descriptive and retrospective evaluation of the 30 years of infestation of the state of São Paulo by the vectors *Ae. aegypti* and *Ae. albopictus* showed nearly total territory coverage (99.69%). With trajectories of initially antagonistic infestation and unequal larval abundances, *Ae. aegypti* has prevailed over *Ae. albopictus* . This performance may be related to behavioral differences and ecological aspects of each species^1 , 13 , 14^ .

The onset of *Ae. aegypti* infestation in São Paulo was observed in the north-western municipalities of the state, taken as a result of the influence of infestations in the states of Mato Grosso do Sul and Paraná^15^ . The first records of *Ae. albopictus* occurred in the southeast of the country, the most densely populated region of Brazil. Such information is important, since it is a space very close to important industrial, commercial and financial complexes of the state and the country. It is an area of important road axes and intense population flow, facilitators in the dissemination of these vectors^16^ . The expansion of *Ae. albopictus* in the Vale do Paraíba region seen in the first 15 years can be ascribed to the absence of *Ae. aegypti* , since they are homologous species and therefore compete for the same ecological niche^1 , 14^ .

Surveillance and control actions of *Ae. aegypti* in developing countries, even in situations where resources for vector control have been appropriate, were often unsuccessful^3 , 4 , 16^ . On the other hand, although *Ae. albopictus* was not a target species for these actions, it was constantly subject to their influence in *Ae. Aegypti* -infested areas, which may have caused selective pressure. The state of São Paulo has recorded its infestation since 1986^5^ , first in areas with high population density and intense population flow, a factor that allowed its dispersion. However, another survey in São Paulo did not demonstrate any relationship between the pattern of geographic expansion and the demographic density of *Ae. albopictus* . The authors suggested the influence of other determining factors^4^ .

The problem of an infestation is localized, and the compartmentalization of the region into municipalities, districts and even blocks favors the success of the control, since each sector or neighborhood of a city can present a reality in the different types of properties and breeding places attended by *Ae. albopictus* and *Ae. aegypti,* which constitute an area of risk for colonization^4 , 17^ . Another study on the influence of different urban strata on the occupation of the city of São Paulo by *Ae. aegypti* showed that the urbanization gradient acts in the population expansion process of this vector, evidencing the influence of the environmental heterogeneity of each stratum in which the mosquito is found^18^ .

The antagonistic trajectories mentioned previously underwent changes in time and space, to the extent that one of the species was established in cities first infested by the other. Thus, the dispersion advanced and culminated in the co-occurrence of *Ae. Aegypti* and *Ae. albopictus* in many municipalities (94%) by 2015. Other authors have already observed the association of these *Stegomyia* in several types of breeding places, mentioning that, although the relation of coexistence in urban and residential green areas is clear, there was a spatial separation of habitats, and suggested the occurrence of interspecific competition for oviposition sites^19 , 20^ . It is noteworthy that here co-occurrence was not a transitory situation, since it remains until the present day.

The dispersion of these vectors was a consequence of the increased global connection, and its distribution was influenced by several factors, among which temperature is indicated as important in the biology and behavior of these species^7 , 15 , 21^ . Our study showed a clear and similar seasonal profile for these species, with presence in every month of the year, but with different larval densities. The highest abundances observed in the summer months coincided with the higher temperatures. Temperature seems to be one of the abiotic factors strictly related to the activities of *Ae. aegypti* . In a study carried out in the state of SP, the authors evidenced the effects of temperature on the geographic expansion of this species, while for *Ae. albopictus* these implications were not clear^15^ .

In Southern Brazil, the preponderance of temperature in the longevity and fecundity of these species was confirmed, with a positive correlation between population increase and average monthly temperature^22 , 23^ . This may be aggravated by the increase in the average temperature of the planet, which in turn should contribute to the expansion in Brazil of the distribution area of such other mosquito-borne viruses as oropouche, mayaro, rocio, and the Saint Louis encephalitis virus^23^ .

In the present study, the increase of the highest average temperature ranges occurred in the northwest-southeast direction, coinciding with the geographical path of *Ae. Aegypti* infestation *,* which may have provided conditions for its expansion in the state. It is deduced that the temperature delayed but did not prevent the dispersion of this vector to the southeast region of the state *,* nor did it establish limits for the dissemination of the species studied.

Analyses of the annual minimum temperature series of SP from 1951 to 2006 showed elevation tendencies^24^ . Similarly, the city of São Paulo has already presented changes in the historical series of temperature, indicating urbanization as a factor that affects the microclimate by creating islands of heat in the city^8 , 25^ .

The entomological surveillance in the state of São Paulo^5^ , based on indicators, allowed the analysis of BI values routinely used to estimate the larval density of these mosquitoes. It was not our purpose here to discuss whether or not this larval index is a good indicator, presumably a limitation of the study, but rather to analyze it, because it has been used since 1985. Although BI mean values have varied over the years and across species, the percentage of containers with *Ae. aegypti* was 4.27 times as high as that of those containing *Ae. albopictus* .

Glasser et al.^26^ , studying the immature forms of these species in Baixada Santista, a region of high population density in the south coast of the state of São Paulo, also observed that *Ae. albopictus* levels were much lower than those of *Ae. aegypti* , and that *Ae. aegypti* was present in most of these containers.

The highest percentage of containers with *Ae. aegypti* observed in our study allows to infer that its larval abundance is greater than that of *Ae. albopictus* , important information about the ecology of these species, since it provides evidence of an active competitive effect between these mosquitoes. This finding requires attention, given the usual sylvatic habits of this species, because their larval co-occurrence occurs in artificial breeding sites very common in areas of great human concentration^20 , 26 - 29^ .

The patterns of relative abundance of the two species may vary widely. Differently from what is reported here, in southern Florida there was a decline in the abundance of *Ae. aegypti* after the invasion of *Ae. albopictus* ^28^ . It is understood that the success of a species may vary depending on the ecological, geographical, environmental and epidemiological circumstances.

The findings of our study revealed a state-wise predominance of *Ae. Aegypti* , but frequent presence of *Ae. albopictus* in densely urbanized areas *.* Although it is predominantly a peridomiciliar species, a possible selection of populations is reiterated based on a trend towards domiciliation ^9^ .

Although with lower densities, the presence of *Ae. albopictus* in these areas increases the risk of transmission of several arboviruses, which in turn reinforces the need to maintain and increase the monitoring of their populations as an essential part of entomological surveillance. These data are unprecedented and, therefore, constitute valuable information that contributes to a better understanding of the dynamics of arboviral transmission, besides providing elements for vector control.
