# Erratum

**DOI:** 10.11606/s1518-8787.2019053001264err

**Published:** 2019-12-16

In “**Vectors of arboviruses in the state of São Paulo: 30 years of Aedes aegypti and Aedes albopictus**” Rev. Saude Publica [online]. 2019, vol.53:84, ISSN 1518-8787, http://dx.doi.org/10.11606/s1518-8787.2019053001264, the RSP corrects the author’s last name.

**Where you read:**

Marcia Moreira Holcmam

**You should read:**

Marcia Moreira Holcman

