# Supplementary material for: Vectors of arboviruses in the state of São Paulo: 30 years of Aedes aegypti and Aedes albopictus
Source: Rev Saude Publica. 2019 Sep 18;53:84. doi: 10.11606/s1518-8787.2019053001264 (PMC6763287; doi:10.11606/s1518-8787.2019053001264)
Supplement: Translation

# Translation: Vetores de arboviroses no estado de São Paulo: 30 anos de *Aedes aegypti* e *Aedes albopictus*

**Authors:** Dalton Pereira da Fonseca, Lígia Leandro Nunes Serpa, Gerson Laurindo Barbosa, Mariza Pereira, Marcia Moreira Holcmam, Júlio Cesar Voltolini, Gisela Rita Alvarenga Monteiro Marques

**Affiliations:** I Superintendência de Controle de Endemias. Departamento de Controle de Vetores. São Paulo, SP, Brasil; II Universidade de Taubaté. Faculdade de Ciências Biológicas. Taubaté, SP, Brasil

**Keywords:** Aedes, Mosquitos Vetores, Vetores de Doenças, Análise Espacial, Controle de Vetores

## Abstract

**OBJETIVO:**

Descrever a infestação dos municípios paulistas pelos vetores *Aedes aegypti* e *Aedes albopictus* , caracterizar a sazonalidade e analisar as temperaturas médias e as densidades larvárias.

**MÉTODOS:**

Foram empregados mapas com informações sobre a infestação dos municípios entre 1986 e 2015. A análise da densidade larvária das espécies pelo teste de Wilcoxon utilizou os valores do índice de Breteau para *Ae. aegypti* e *Ae. albopictus* obtidos no banco de dados da Superintendência de Controle de Endemias. Na descrição sazonal, foram calculadas as médias aritméticas de cada um por mês e ano. As análises de temperaturas médias foram apresentadas em mapas com gradientes de cores.

**RESULTADOS:**

O estado de São Paulo apresenta-se na atualidade quase totalmente infestado, com coocorrência das espécies em 93,64% dos municípios. A análise da sazonalidade evidenciou o primeiro trimestre como o período mais favorável para a abundância larval. Observou-se elevação das temperaturas médias em áreas geográficas coincidentes com a trajetória temporal da expansão territorial de *Ae. aegypti* . O valor médio da densidade larvária de *Ae. aegypti* foi maior do que o verificado para *Ae. albopictus* (p = 0,00).

**CONCLUSÕES:**

Esses culicídeos apresentaram, inicialmente, ocupação em áreas distintas e opostas. Entretanto, no decorrer do tempo, a coocorrência mostrou quão grande é a capacidade de adaptação, mesmo em face de conjunturas sociais e urbanas diferentes. A elevação da temperatura média contribuiu para a expansão geográfica de *Ae. aegypti* , assim como para o perfil nitidamente sazonal de ambas as espécies. No geral, a infestação larvária por *Ae. aegypti* foi predominante, o que evidenciou sua superioridade competitiva. Tais dados propiciam maior entendimento da dinâmica de transmissão de arboviroses no estado de São Paulo e são subsídios para a vigilância e o controle desses vetores.

## INTRODUÇÃO

*Aedes* ( *Stegomyia* ) *aegypti* e *Aedes* ( *Stegomyia* ) *albopictus* são importantes vetores de vírus causadores de doenças emergentes e reemergentes, como dengue, Zika, chikungunya e febre amarela, e apresentam ampla distribuição geográfica global. A expansão geográfica desses mosquitos no mundo é influenciada por diferentes fatores e tem sido acompanhada, em alguns locais, pela diminuição de sua abundância, eliminação do outro vetor, ou pela coexistência em extensas regiões da América^1^ . No Brasil, essas espécies foram introduzidas em diferentes momentos: *Ae. aegypti* no período da colonização e *Ae. albopictus* no final do século XX^2 , 3^ .

No estado de São Paulo (ESP), no início da década de 1980, foi detectada a presença do *Ae. aegypti* na região portuária de Santos. Passados cinco anos, após amplo levantamento entomológico, foi identificada infestação domiciliar em nove municípios, todos situados na região noroeste do estado^3 , 4^ . Na sequência, em 1987, focos de *Ae. albopictus* foram registrados na região leste do ESP^5^ . A detecção dessas espécies desencadeou ações específicas de controle; contudo, os esforços mantêm-se direcionados na tentativa de manter baixos os índices de infestação desses vetores^6^ .

A dispersão desses mosquitos provavelmente foi favorecida pelo aumento da circulação de pessoas e intensificação de atividades comerciais, fatores que agravam o risco de arboviroses, mesmo em regiões indenes^7^ . Esse quadro pode ainda ter sido intensificado pelo efeito das mudanças climáticas, o que por sua vez provoca impacto na distribuição desses transmissores^8^ .

A presença desses mosquitos nos municípios paulistas tem sido identificada, nos últimos anos, de maneira crescente e acelerada, perfil que impõe dificuldades para os programas e ações de controle desses vetores. Isso reforça a importância da investigação entomológica, que pode ajudar a definir os limites espaciais da transmissão de vários arbovírus e compreender melhor seus processos ecológicos e de ocupação no espaço territorial, ao longo do tempo. Assim, o conhecimento da distribuição espaço-temporal e abundância dessesvetores constituem importantes objetivos da vigilância entomológica, pois permitem monitorar seu comportamento populacional, essencial para o planejamento em ações de saúde pública. Diante do exposto, pretendeu-se descrever a expansão geográfica de *Ae. aegypti e* de *Ae. albopictus* nos municípios paulistas, supondo-se que suas densidades populacionais sejam diferentes.

## MÉTODOS

Trata-se de um estudo descritivo e retrospectivo da infestação do estado de São Paulo por *Ae. aegypti* e *Ae. albopictus,* de 1986 a 2015 *.* A área de estudo constitui-se no mais populoso estado da federação, com aproximadamente 44,85 milhões de habitantes e densidade populacional de 168 habitantes por km^2^. Localizado na região Sudeste do país, faz divisa com os estados do Paraná, Mato Grosso do Sul, Minas Gerais e Rio de Janeiro. É um dos mais importantes polos de desenvolvimento do Hemisfério Sul, representando 3% da superfície do Brasil, embora sua influência econômica ultrapasse muito seus limites territoriais^9^ . A classificação climática abrange sete tipos, a maioria de clima úmido. O tipo dominante é caracterizado pelo clima tropical de altitude, com chuvas no verão, seca no inverno e temperatura média anual de 20 °C a 22 °C, sendo a temperatura média do mês mais quente superior a 22 °C^10^ .

O processo de transformação do espaço geográfico paulista sofreu, ao longo dos últimos anos, transformações socioeconômicas acompanhadas por um intenso processo de redistribuição da população humana, o que resultou em uma concentração regionalmente diferenciada^11^ . O indicador entomológico analisado foi o índice de Breteau (IB), adotado para o acompanhamento das atividades de vigilância e controle, que possibilita a obtenção dos níveis de infestação larvária por *Ae. aegypti* e por *Ae. albopictus* para cada município^6^ .

$$\mathrm{IB}\;=\;\frac{nº\;\mathrm{de}\;\mathrm{recipientes}\;\mathrm{positivos}\;\mathrm{para}\;a\;\mathrm{presença}\;\mathrm{da}\;\mathrm{espécie}}{nº\;\mathrm{de}\;\mathrm{imóveis}\;\mathrm{pesquisados}}\;x\;100$$

Os dados foram obtidos do sistema de informação on-line da Superintendência de Controle de Endemias (Sucen), compartilhado pelo estado e seus municípios. Os valores do IB mensuram os criadouros por meio da contagem dos recipientes com larvas de cada espécie em grupos de 100 imóveis pesquisados. O valor obtido indica a intensidade da infestação domiciliar, o que possibilita a estimativa da densidade.

A descrição dos municípios infestados para cada uma das espécies, no tempo e no espaço, foi apresentada em mapas anuais. Município infestado é aquele no qual a espécie apresenta-se estabelecida, ou seja, presente mesmo após a realização de ações de controle, com registros de seu desenvolvimento e reprodução, albergando em sua extensão formas imaturas desses vetores^3^ .

Para caracterizar a sazonalidade e analisar as densidades populacionais, foram calculadas as médias aritméticas dos valores de IB de cada uma das espécies por mês e ano do período de estudo. Os dados foram tratados pelo software Bioestat 5.3, comparados pelo teste de Wilcoxon ( *Wilcoxon matched pairs test* ou *Wilcoxon signed-ranks test* ). Foram considerados significativos os valores de p < 0,05.

Para analisar as temperaturas médias no estado de São Paulo, foram utilizados dados das estações meteorológicas do Banco de Dados Meteorológicos para Ensino e Pesquisa, do Instituto Nacional de Meteorologia^13^ . Os dados foram apresentados em mapas para os anos de 1986, 1990, 1995, 2000, 2005, 2010 e 2015. As cores representaram os diferentes gradientes de temperatura, com o maior deles indicado pelo tom vermelho escuro e o menor pelo mais claro.

## RESULTADOS

A série histórica de 30 anos da infestação dos municípios paulistas por *Ae. aegypti* ou *Ae. albopictus* é apresentada na Tabela 1 . Entre os anos de 1986 e 1996, pode-se verificar o aumento do número de municípios, passando de 572 para 645, número que se mantém até os dias atuais.

**Tabela 1:** Distribuição do número de municípios infestados por *Ae. aegypti* ou *Ae. Albopictus* . Estado de São Paulo, Brasil, 1986–2015.

Número de municípios						

Ano	Existentes	Infestados por *Ae. aegypti*	Infestados por *Ae. albopictus*	Infestação mista*	Total de infestados	% com infestação
1986	572	65	0	0	65	11,36
1987	572	133	6	0	139	24,30
1988	572	186	19	0	205	35,84
1989	572	227	23	6	256	44,76
1990	572	257	38	35	330	57,69
1991	582	282	48	48	378	64,95
1992	625	262	91	88	441	70,56
1993	625	227	147	159	533	85,28
1994	637	170	174	236	580	91,05
1995	637	162	178	255	595	93,41
1996	645	105	179	321	605	93,80
1997	645	77	182	368	627	97,21
1998	645	74	165	389	628	97,36
1999	645	73	154	401	628	97,36
2000	645	72	150	406	628	97,36
2001	645	72	142	414	628	97,36
2002	645	48	132	450	630	97,67
2003	645	42	126	462	630	97,67
2004	645	40	124	466	630	97,67
2005	645	40	117	473	630	97,67
2006	645	40	107	483	630	97,67
2007	645	39	96	495	630	97,67
2008	645	39	87	504	630	97,67
2009	645	37	74	519	630	97,67
2010	645	37	59	535	631	97,83
2011	645	39	36	566	641	99,38
2012	645	38	34	569	641	99,38
2013	645	38	22	581	641	99,38
2014	645	36	14	591	641	99,38
2015	645	35	4	604	643	99,69

* Infestados por ambas espécies.

A infestação por *Ae. aegypti* teve início em 1986, enquanto a por *Ae. albopictus* , em 1987, em áreas geográficas distintas e opostas. No primeiro ano, a dispersão desses *Aedes* atingiu respectivamente 133 (23,25%) e seis (1,05%) dos municípios existentes à época. Nos anos seguintes, observa-se um aumento do número de municípios infestados por uma ou outra espécie, com maior dispersão de *Ae. aegypti* e crescimento superior em relação ao *Ae. albopictus* . A infestação do número de municípios na primeira metade do estudo (1986 a 2000) foi maior do que na segunda metade. Em 1994, a infestação alcançou o patamar de 91,05%. No último ano do estudo, 2015, quase a totalidade do território paulista (99,69%) encontrava-se infestado.

Já a coocorrência desses *Stegomyia* , ou seja, infestação mista, foi constatada em seis municípios em 1989, elevando-se para 37,05% em 1994, momento a partir do qual o número de municípios infestados exclusivamente por *Ae. albopictus* também aumenta, porém em área geográfica diferente da ocupada por *Ae. aegypti* . A infestação mista, embora tenha se modificado ao longo do tempo e de região para região, passou a dominar o espaço geográfico estadual, sendo registrada em 604 (93,64%) dos municípios existentes. Dos 41 municípios restantes, 35 ainda registravam infestação exclusiva por *Ae. aegypti* e seis por *Ae. albopictus.* Localizados em áreas geográficas diferentes, os municípios infestados por *Ae. aegypti* encontravam-se na região centro-oeste paulista, enquanto os infestados por *Ae. albopictus* , na Região da Grande São Paulo e Vale do Paraíba. Apenas os municípios de Campos do Jordão, na região do Vale do Paraíba, e de Ribeirão Grande, na região de Sorocaba, não registravam infestação por essas espécies.

A Figura 1 exibe a trajetória geográfica dessa infestação. Verifica-se que a ocupação não se deu de forma homogênea no tempo e espaço, porém foi de rápida expansão geográfica. No primeiro ano, 1986, observa-se o estabelecimento de *Ae. aegypti* na região noroeste, metade superior do ESP. No ano seguinte, 1987, têm-se os primeiros registros de *Ae. albopictus,* em municípios situados às margens da rodovia Presidente Dutra (BR-116), no sentido RJ-SP. Essa é uma área denominada Vale do Paraíba, na região leste do ESP, oposta à ocupada por *Ae. aegypti* . Na sequência, em 1988, aquela espécie estabeleceu-se em número reduzido de municípios na região nordeste do estado. A coocorrência dessas espécies foi registrada na região norte-nordeste, até então ocupada somente por *Ae. albopictus* .

**Figura 1:** Municípios infestados por *Ae. aegypti,* por *Ae* . *albopictus* e por ambas espécies. Estado de São Paulo, 1986–2015.

Nos anos que se seguiram, observa-se grande ampliação da área infestada, de maneira que, na primeira metade da década de 1990, foi possível diferenciar três padrões na expansão geográfica das espécies. Mais a noroeste do estado, a infestação deu-se por *Ae. Aegypti* ; a sul-sudeste, por *Ae. albopictus* ; no sentido nordeste a sudoeste, uma faixa central, com infestação mista.

Cronologicamente, a coocorrência avançou mais rápido no sentido noroeste-oeste, do que na região sul-sudeste, até então ocupada somente por *Ae. albopictus* . Tal quadro não se manteve por muito tempo, já que, a partir de 2001, o *Ae. aegypti* expandiu-se por quase toda a Região do Vale do Paraíba, última do estado a se infestar por essa espécie. Como é possível observar, em 2015 poucos municípios permanecem sem o registro da coocorrência das espécies.

A Figura 2 apresenta os valores médios mensais de IB para *Ae. aegypti* e para *Ae. albopictus* , nos 30 anos de estudo. Observa-se um comportamento sazonal semelhante, porém com valores de densidades larvárias diferentes. Embora com frequências distintas, observa-se o registro das espécies em todos os meses do ano. Os resultados mostraram que a maior abundância dos vetores foi no primeiro trimestre, meses de verão (janeiro a março). No segundo trimestre (abril a junho), observa-se queda dos índices de densidade das duas espécies e na sequência, meses de julho a outubro, *Ae. albopictus* em valores bastante reduzidos. Por outro lado, *Ae. aegypti* reduz sua densidade até agosto (valores de IB próximos de 1,0), porém retoma seu crescimento populacional nos demais meses do ano (outubro a dezembro), com índices médios próximos a 3,0.

**Figura 2:** Distribuição mensal dos valores médios de índice de Breteau (IB) de *Ae. aegypti* e de *Ae. Albopictus.* Estado de São Paulo, 1986–2015.

A Figura 3 mostra o perfil da temperatura média em grau Celsius (°C) para o estado de São Paulo. Os sete mapas mostram a sequência cronológica de ampliação da faixa geográfica com temperaturas médias mais elevadas. Observa-se que essa elevação é crescente ao longo dos anos, deslocando-se para o interior do estado, a partir do alinhamento planáltico, e para o litoral, junto às serras de Paranapiacaba, do Mar e da Mantiqueira.

**Figura 3:** Perfil das temperaturas médias, segundo ano. Estado de São Paulo, Brasil, 1986, 1990, 1995, 2000, 2005, 2010 e 2015.

Os valores médios do IB são apresentados na Tabela 2 . Observa-se que variaram segundo ano e espécie; todavia, os indicadores de *Ae. aegypti* sempre foram superiores aos verificados para *Ae. albopictus* . No período de estudo foram realizadas, em média, 416 avaliações de densidade larvária por ano, resultado de 26.910.205 imóveis pesquisados no período de estudo.

**Tabela 2:** Distribuição anual dos valores médios do índice de Breteau (IB) e percentagem de recipientes com cada espécie. Estado de São Paulo, Brasil, 1986–2015.

Ano	IB *Ae. aegypti*	IB *Ae. albopictus*	IB total	% de recipientes com *Ae. aegypti*	% de recipientes com *Ae. albopictus*
1986	2,28	0,01	2,29	99,56	0,44
1987	2,18	0,01	2,19	99,54	0,46
1988	2,73	0,19	2,92	93,52	6,48
1989	2,96	0,10	3,06	96,68	3,32
1990	3,60	0,19	3,79	94,98	5,02
1991	1,84	0,21	2,05	89,59	10,41
1992	2,35	0,44	2,79	84,20	15,80
1993	2,76	0,02	2,79	99,18	0,82
1994	2,40	1,61	4,02	59,87	40,13
1995	2,30	1,55	3,85	59,77	40,23
1996	3,68	1,38	5,07	72,68	27,32
1997	3,17	1,03	4,20	75,48	24,52
1998	3,42	1,42	4,83	70,72	29,28
1999	2,39	0,91	3,30	72,52	27,48
2000	3,43	1,53	4,96	69,25	30,75
2001	2,34	0,97	3,31	70,71	29,29
2002	1,15	0,45	1,60	72,14	27,86
2003	1,23	0,39	1,62	75,96	24,04
2004	1,54	0,40	1,95	79,28	20,72
2005	1,98	0,33	2,30	85,81	14,19
2006	1,59	0,22	1,80	87,96	12,04
2007	1,38	0,20	1,58	87,31	12,69
2008	1,02	0,17	1,19	85,75	14,25
2009	1,41	0,14	1,54	91,09	8,91
2010	0,98	0,21	1,19	82,50	17,50
2011	0,97	0,15	1,11	86,82	13,18
2012	1,08	0,11	1,19	90,65	9,35
2013	1,26	0,14	1,40	90,30	9,70
2014	1,05	0,09	1,13	92,38	7,62
2015	1,11	0,12	1,23	89,87	10,13
Média	2,05	0,48	2,54	83,54	16,46
Erro-padrão	0,16	0,11	0,23	2,06	2,06

A média do IB dos 30 anos de estudo foi de 2,05, com erro padrão (EP) = 0,16, e 0,48 (EP = 0,11) para *Ae. aegypti* e para *Ae. albopictus* , respectivamente. Do total de recipientes pesquisados, a presença da primeira espécie foi registrada em 83,54% e a da segunda em 16,46%. A comparação dos valores médios de IB de *Ae. aegypti* e de *Ae. albopictus* mostrou diferença estatística significativa (n = 30; T = 0,00; Z = 4,78; p < 0,001), o que indica que a densidade larvária média da primeira foi nitidamente superior à verificada para a segunda espécie, 4,27 vezes maior, se traduzindo na maior percentagem de recipientes positivos com presença de formas imaturas de *Ae. aegypti* .

## DISCUSSÃO

A avaliação descritiva e retrospectiva dos 30 anos da infestação do estado de São Paulo por vetores *Ae. aegypti* e *Ae. albopictus* mostrou abrangência praticamente total do território (99,69%). Com trajetórias de infestação inicialmente antagônicas e abundâncias larvárias desiguais, *Ae. aegypti* predominou em relação ao *Ae. albopictus* . Esse desempenho pode estar relacionado às diferenças de comportamento e de aspectos ecológicos de cada uma das espécies^1 , 13 , 14^ .

O início da infestação por *Ae. aegypti* no estado de São Paulo foi observado nos municípios a noroeste do estado, apontada como resultante da influência de infestações nos estados do Mato Grosso do Sul e do Paraná^15^ . Opostamente, os primeiros registros de *Ae. albopictus* se deram no Sudeste do pais, região onde se concentra o maior contingente populacional do Brasil. Tal informação é importante, uma vez que se trata de espaço muito próximo de importantes complexos industriais, comerciais e financeiros do estado e do país. É uma área de importantes eixos rodoviários e intenso fluxo populacional, facilitadores na disseminação desses vetores^16^ . A expansão de *Ae. albopictus* na região do Vale do Paraíba, nos primeiros 15 anos analisados, pode ser atribuída à ausência de *Ae. aegypti* , uma vez que são espécies homólogas e, portanto, disputam o mesmo nicho ecológico^1 , 14^ .

As ações de vigilância e controle de *Ae. aegypti* nos países em desenvolvimento, mesmo em situações em que os recursos destinados ao controle do vetor tenham sido apropriados, muitas vezes não alcançaram o sucesso desejado^3 , 4 , 16^ . Por outro lado, embora *Ae. albopictus* não tenha sido espécie-alvo dessas ações, esteve constantemente sujeito a sua influência em áreas infestadas por *Ae. aegypti* , o que possivelmente pode ter provocado pressão seletiva. O estado de São Paulo registra sua infestação desde 1986^5^ , inicialmente em áreas com elevada densidade demográfica e intenso fluxo populacional, fator que possibilitou sua dispersão. Todavia, outra pesquisa em São Paulo não demonstrou a relação entre o padrão de expansão geográfica e a densidade demográfica na dispersão de *Ae. albopictus* . Os autores sugeriram a influência de outros fatores determinantes^4^ .

O problema de uma infestação é localizado, e a compartimentalização da região em municípios, distritos e até mesmo quadras propicia o sucesso do controle, pois cada setor ou bairro de uma cidade pode apresentar uma realidade nos diferentes tipos de imóveis e criadouros frequentados por *Ae. albopictus* e *Ae. aegypti,* que constituem área de risco para a colonização^4 , 17^ . Outro estudo sobre a influência de diferentes estratos urbanos na ocupação da cidade de São Paulo, SP, por *Ae. aegypti* mostrou evidências de que o gradiente de urbanização atua no processo de expansão populacional desse vetor, evidenciando a influência da heterogeneidade ambiental de cada estrato no qual o mosquito é encontrado^18^ .

As trajetórias antagônicas mencionadas anteriormente sofreram alterações no tempo e espaço, na medida em que uma das espécies se estabelecia em cidades primeiramente infestadas pela outra. Assim, a dispersão avançou e culminou na coocorrência de *Ae. aegypti* e *Ae. albopictus* em grande número de municípios (94%) em 2015. Outros autores já observaram a associação desses *Stegomyia* em diversos tipos de criadouros, mencionando que, embora seja clara a relação de coexistência em áreas verdes urbanas e residenciais, foi observada uma separação espacial de hábitats, e sugeriram a ocorrência da competição interespecífica por sítios de oviposição^19 , 20^ . Vale ressaltar que aqui a coocorrência não foi uma situação transitória, uma vez que permanece até os dias atuais.

A dispersão desses vetores foi consequência do aumento da conexão global, e sua distribuição foi influenciada por vários fatores, dentre os quais a temperatura é apontada como importante na biologia e comportamento dessas espécies^7 , 15 , 21^ . Nosso estudo mostrou perfil sazonal nítido e semelhante para as espécies, com presença em todos os meses do ano, porém com densidades larvárias diferentes. As maiores abundâncias verificadas nos meses de verão foram coincidentes com as temperaturas mais elevadas. A temperatura parece ser um dos fatores abióticos estritamente relacionados às atividades de *Ae. aegypti* . Num estudo realizado no ESP, os autores evidenciaram os efeitos da temperatura na expansão geográfica dessa espécie, enquanto para *Ae. albopictus* tais implicações não ficaram claras^15^ .

No Sul do Brasil, a preponderância da temperatura na longevidade e fecundidade dessas espécies foi comprovada, com correlação positiva entre o aumento populacional e a temperatura média mensal^22 , 23^ . Isso pode ser agravado mediante o aumento da temperatura média do planeta que, por sua vez, deve contribuir para ampliar no Brasil a área de distribuição de outros vírus transmitidos por mosquitos, como oropouche, mayaro, rocio e o vírus da encefalite de Saint Louis^23^ .

No presente estudo, a ampliação das faixas de temperaturas médias mais elevadas ocorreu no sentido noroeste-sudeste, direção coincidente com a trajetória geográfica de infestação do *Ae. aegypti,* o que pode ter propiciado condições para sua expansão no estado. Deduz-se que a temperatura retardou a dispersão desse vetor para a região sudeste do estado *,* porém não a impediu e tampouco estabeleceu limites para a disseminação das espécies estudadas.

Análises das séries anuais de temperatura mínima do ESP de 1951 a 2006 mostraram tendências de elevação^24^ . No mesmo sentido, a cidade de São Paulo já apresenta alteração na série histórica de temperatura, indicando a urbanização como fator que afeta o microclima por criar ilhas de calor na cidade^8 , 25^ .

A vigilância entomológica no estado de São Paulo^5^ , estruturada a partir de indicadores, permitiu análise dos valores de IB, rotineiramente empregados para estimar a densidade larvária desses mosquitos. Não constituiu propósito discutir se o referido índice larval é ou não bom indicador, presumível limitação do estudo, mas sim analisá-lo, uma vez que é utilizado desde 1985. Embora os valores médios do IB tenham variado ao longo dos anos e entre as espécies, constatou-se que a percentagem de recipientes com larvas de *Ae. aegypti* foi 4,27 vezes maior que a de *Ae. albopictus* .

Glasser et al.^26^ , estudando formas imaturas dessas espécies na Baixada Santista, região de elevada densidade demográfica do litoral sul do estado de São Paulo, também observaram que os níveis de infestação de *Ae. albopictus* eram muito inferiores aos encontrados para *Ae. aegypti* , e que *Ae. aegypti* estava presente na maioria desses recipientes.

A maior percentagem de recipientes com *Ae. aegypti* observada em nosso estudo permite inferir que sua abundância larvária se encontra superior à de *Ae. albopictus,* informação importante sobre a ecologia dessas espécies, uma vez que fornece evidências de um efeito competitivo atuante entre esses mosquitos. Esse achado requer atenção, dados os hábitos semisselváticos desta espécie, pois sua coocorrência larval se dá em criadouros artificiais muito comuns em áreas de grande concentração humana^20 , 26^ .

Os padrões de abundância relativa das duas espécies podem variar amplamente. Diferentemente do observado no presente estudo, no sul da Flórida (EUA), foi verificado declínio na abundância de *Ae. aegypti* após a invasão de *Ae. albopictus* ^28^ . Assim entende-se que, dependendo das circunstâncias ecológicas, geográficas, ambientais e epidemiológicas, o êxito de uma espécie pode variar.

Os achados do nosso estudo revelaram perfil estadual de predomínio de *Ae. aegypti* , porém com frequente presença de *Ae. albopictus* em áreas densamente urbanizadas *.* Em que pese tratar-se de espécie predominantemente peridomiciliar, reitera-se uma possível seleção de populações com base numa evolução para a domiciliação^29^ .

Embora com menores densidades, a presença de *Ae. albopictus* nessas áreas aumenta o risco de transmissão de vários arbovírus, o que, por sua vez, reforça a necessidade da manutenção e incremento do monitoramento de suas populações como parte imprescindível da vigilância entomológica. Esses dados são inéditos e, portanto, constituem informação valiosa que contribui para o melhor entendimento da dinâmica de transmissão de arboviroses, além de oferecer subsídios para o controle de vetores.
