# Supplementary material for: Erratum
Source: Rev Saude Publica. 2019 Dec 16;53:84err. doi: 10.11606/s1518-8787.2019053001264err (PMC6919285; doi:10.11606/s1518-8787.2019053001264err)
Supplement: Translation

# Translation: Errata

No artigo. “**Vetores de arboviroses no estado de São Paulo: 30 anos de Aedes aegypti e Aedes albopictus**” Rev. Saude Publica [online]. 2019, vol.53:84, ISSN 1518-8787, http://dx.doi.org/10.11606/s1518-8787.2019053001264, a RSP corrige o sobrenome do autor.

**Onde se lia:**

Marcia Moreira Holcmam

**Leia-se:**

Marcia Moreira Holcman
